# Tentative T Cells: Memory Cells Are Quick to Respond, but Slow to Divide

**DOI:** 10.1371/journal.ppat.1000041

**Published:** 2008-04-11

**Authors:** Jason K. Whitmire, Boreth Eam, J. Lindsay Whitton

**Affiliations:** Molecular and Integrative Neurosciences Department, The Scripps Research Institute, La Jolla, California, United States of America; National Institutes of Health-NIAID, United States of America

## Abstract

T cell memory is a cornerstone of protective immunity, and is the key element in successful vaccination. Upon encountering the relevant pathogen, memory T cells are thought to initiate cell division much more rapidly than their naïve counterparts, and this is thought to confer a significant biological advantage upon an immune host. Here, we use traceable TCR-transgenic T cells to evaluate this proposed characteristic in CD4^+^ and CD8^+^ memory T cells. We find that, even in the presence of abundant antigen that was sufficient to induce *in vivo* IFNγ production by memory T cells, both memory and naïve T cells show an extended, and indistinguishable, delay in the onset of proliferation. Although memory cells can detect, and respond to, virus infection within a few hours, their proliferation did not begin until ∼3 days after infection, and occurred simultaneously in all anatomical compartments. Thereafter, cell division was extraordinarily rapid for both naïve and memory cells, with the latter showing a somewhat accelerated accumulation. We propose that, by permitting memory T cells to rapidly exert their effector functions while delaying the onset of their proliferation, evolution has provided a safeguard that balances the risk of infection against the consequences of severe T cell–mediated immunopathology.

## Author Summary

Vaccines are the only medical products that should be administered to almost every human worldwide, and it is well-known that they act by inducing so-called “memory” cells that can protect against subsequent encounter with the related micro-organism. Surprisingly, we do not understand precisely how these memory cells work: in what way are they better than non-memory (“naïve”) cells, and how do they exert their life-saving functions? It was thought that, following exposure to the relevant microbial antigen, memory cells responded by increasing in number much more quickly than naïve cells and that they achieved this greater abundance by two means: first, by initiating cell division sooner after infection; and, second, by multiplying more rapidly thereafter. Here, we show that neither is true. Memory cells, like naïve cells, begin to divide only after lengthy (2–3 day) delay after virus infection, and their subsequent rate of division is no faster than that of naïve cells. We speculate on the possible evolutionary benefits that might accrue from this lengthy delay before cell division begins.

## Introduction

After virus infection, a small number of naïve virus-specific T cells begins a process of cell division and differentiation that results in the accumulation of a large number of effector T cells. These cells become sufficiently numerous at around day 5 (for CD8^+^ T cells) or day 6 (for CD4^+^ T cells) to allow their detection by flow cytometry, and their numbers peak at ∼7–10 days after infection. Thereafter, cell numbers decline, and by ∼15 days post-infection the majority of cells remaining are of memory phenotype. These memory T cells are central to the protective immunity that is induced by infections and by vaccination, and are thought to confer several benefits upon the immune host. When compared to naïve cells, memory T cells can be triggered by lower levels of antigen, and they more rapidly express several effector functions [1]–[3]. Furthermore, in contrast to naive T cells, memory T cells efficiently enter non-lymphoid tissues to survey for antigen, facilitating the early detection of, and rapid response to, infection [4],[5]. An additional benefit of memory T cells is their more rapid accumulation after antigen re-exposure. This has been attributed, in large part, to their more rapidly initiating cell division following antigen contact [1],[2].

Memory T cell responses have most commonly been measured in immune mice, where pre-existing memory T cells and antibody could affect the response. To circumvent this concern, many investigators have employed heterologous infections where mice are given one pathogen, which expresses a particular epitope, to induce memory T cell formation, and the mice are subsequently challenged with a different pathogen that expresses that same epitope. In this way, the response of the epitope-specific memory T cells can be measured in the absence of extensive pre-existing memory T cell or antibody responses the secondary pathogen. However, the inflammatory signals during the primary and during the secondary response could vary with the pathogen and the response of memory T cells to heterologous challenge could be different from the response following re-exposure to the original pathogen. Furthermore, in many cases, memory cell responses in immune mice have been compared to the responses of naïve cells in naïve mice (i.e., to classical primary T cell responses); but under these circumstances, any differences observed between the memory and the naïve cells may be due not only to intrinsic differences between the two cell types, but also to differences in the immune environment in which the two populations reside.

It is important to determine the extent to which the faster response is due to the intrinsic, epigenetic changes that are present within memory T cells and how much is due to extrinsic changes that are related to the immune host. These concerns can be largely circumvented by carrying out adoptive transfer experiments and herein, focusing mainly on virus-specific CD4^+^ T cells, we have re-evaluated the antigen responsiveness of naïve and memory T cells, by comparing the organism-wide kinetics of both cell types in the same host animals during the first few days of a viral infection. TCR-transgenic naïve and/or memory T cells were adoptively transferred into mice; experiments were designed to allow us to compare the responses of the two populations very early after infection (by transferring relatively large numbers of cells) or later post-infection (by transferring fewer cells). Using these traceable T cells, we have compared the rates of accumulation of naïve and memory CD4^+^ T cells in lymphoid and non-lymphoid tissues, and determined whether any differences are due to the more rapid initiation of cell division by the memory cells.

## Results

### Early kinetics of naive T cell accumulation and cell division after infection

**Figure 1:**
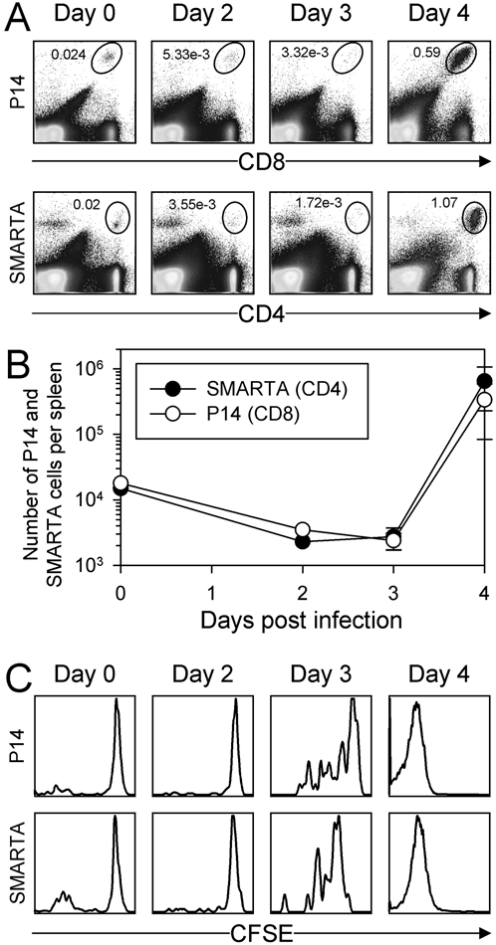
Naive antiviral CD4^+^ and CD8^+^ T cell division has a lag phase of 2–3 days. Equal numbers of CFSE-labeled P14 cells (TcR-transgenic CD8^+^ T cells specific for LCMV GP_33–41_, expressing Thy1.1) and CFSE-labeled SMARTA cells (TcR transgenic CD4^+^ T cells specific for LCMV GP_61–80_, expressing Ly5a) were pooled, and inoculated into wildtype C57BL/6 mice, which then were infected with LCMV. A. At the indicated times after infection, each donor population was identified by flow cytometry (ovals). B. The numbers of P14 and SMARTA T cells in the spleen are shown (mean±SE) at the indicated times after infection (two separate experiments, two mice per experiment). C. After gating to identify the P14 or the SMARTA T cells, the histograms show these cells' CFSE fluorescence. Note that both T cell subsets begin proliferating at the same time (day 3).

The CD4^+^ T cell response to LCMV is non-linear and includes an early period where there is minimal T cell accumulation [6]. To better characterize this early stage of the response for naïve CD4^+^ T cells, and to determine whether a similar pattern is found for naive CD8^+^ T cells, mice were given equal numbers of pooled naïve CFSE-labeled P14 and SMARTA T cells, which can be distinguished from host cells, and from each other, by their expression of congenic T cell markers (Thy1.1 and Ly5a, respectively). The TCR-Tg cells were allowed to engraft for several days, then some of the mice were inoculated with LCMV, and the abundance of the transferred cells was followed daily by flow cytometry. Representative data from individual mice are shown in Figure 1A. A very small percentage of P14 CD8^+^ T cells was found in the spleen in uninfected mice (day 0), and this percentage remained very small through day 3 after infection, but it changed dramatically by day 4. A similar pattern was found for SMARTA CD4^+^ T cells in the same mice, replicating what we have reported before for CD4 T cells [6]. The numbers of P14 CD8^+^ T cells and SMARTA CD4^+^ T cells were determined, and are shown in Figure 1B. There was a slight dip in the number of both cell types at day 2, as has been reported by others [7],[8], and which has been attributed to type I IFN-mediated apoptotic deletion of cells [9] or their retention on DC [8], although the data shown are gated on all isolated live splenocytes, which would include DC. Nevertheless, both CD8 and CD4 T cells show a delay in accumulation that lasts 2–3 days. At day 4 post-infection, however, the cell numbers had increased explosively; both CD4^+^ and CD8^+^ T cells had increased in abundance by >100-fold, indicative of the cells' having divided at least 6–7 times in the ∼24 hour period between sample harvests.

Several hypotheses might be advanced to explain the lack of CD4^+^ and CD8^+^ cell accumulation in the spleen prior to day 4: for example, minimal cell division, or immediate egress of daughter cells from the spleen. To begin to address this issue, we assessed the CFSE fluorescence of the naïve P14 CD8^+^ T cells and SMARTA CD4^+^ T cells in the spleen (Figure 1C). For the first two days there was no loss of CFSE, and on day 3 there was limited cell division; 24 hours later, the cells had divided beyond the limits of detection of the CFSE assay (>7–8 cell divisions). Thus, for naïve cells, the lag phase appears to be related to delayed cell division; once the cells begin to divide, they do so very rapidly, and this coincides with the increase in the cell abundance. Moreover, the pattern holds for both CD4^+^ and CD8^+^ T cells. Note that the presence of T cells of a CFSE-intermediate phenotype at day 3 is most consistent with the cells' actively dividing within the spleen; this conclusion is supported by additional data, below.

### T cell division is initiated synchronously in various lymphoid and non-lymphoid tissues

**Figure 2 ppat-1000041-g002:**
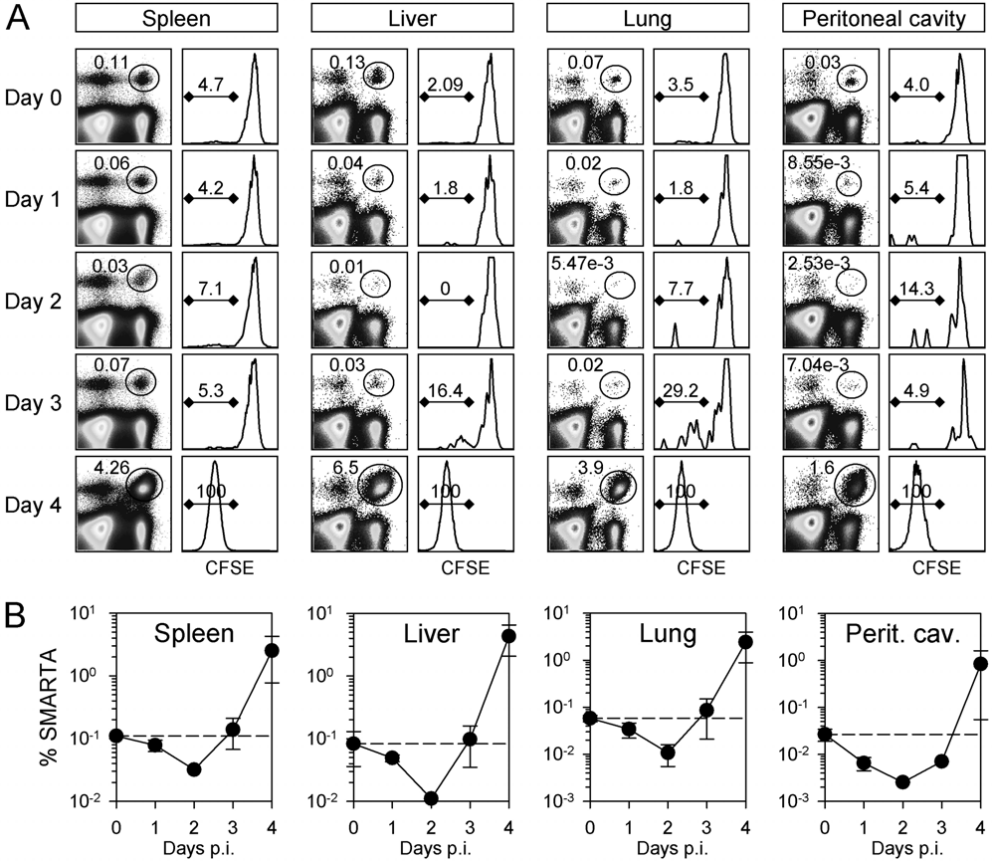
The delay in naïve T cell division is organism-wide. Mice containing approximately 1.4×10^5^ CFSE-labeled SMARTA CD4^+^ T cells were infected with LCMV. At the indicated times after infection, lymphocytes were isolated (2 mice per time point) and the donor cells were identified by flow cytometry. A. The ovals in the dot plots identify the SMARTA CD4^+^ T cells, and the numbers indicate their percentage among leukocytes isolated from each tissue. The histograms show the CFSE-fluorescence of the SMARTA CD4^+^ T cells; the numbers indicate the percentages of SMARTA CD4^+^ T cells that have divided. B. The line graphs show the percentages of SMARTA CD4^+^ T cells among all isolated leukocytes at various times after infection. For each tissue, the dashed line indicates the number of SMARTA cells in uninfected mice.

As noted above, the lag phase observed in the spleen could result from the flight of dividing cells from that organ. Furthermore, the sudden increase in cell number in the spleen at day 4 could be explained by the converse–the rapid recruitment into the spleen of cells that have undergone cell division at some other location. It is, therefore, important to evaluate the kinetics of cell accumulation and cell division in other anatomical sites. Mice containing naïve CFSE-labeled SMARTA cells were infected with LCMV and, at early times after infection, lymphocytes were isolated from the spleen, liver, lung, and peritoneal cavity (Figure 2A). The patterns of cell accumulation (left columns) and CFSE dilution (right columns) in the non-lymphoid tissues were similar to that observed in the spleen; the onset of T cell accumulation was delayed, and the number of cells increased rapidly between day 3 and day 4. Cell division appeared to begin at or after day 3, and by day 4 the cells had divided beyond the limits of the assay in all tissues. The overall pattern of SMARTA CD4^+^ T cell abundance in the spleen mirrored that in other organs (Figure 2B); there was a slight loss of cells early on, and the frequency of cells at day 3 was similar to that in uninfected mice (dashed lines). Significant increases in the abundance of CD4 T cells occurred only after day 3. These data indicate that there is an organism-wide delay in proliferation, which is underscored by the predominance of undivided cells at day 3 in the peritoneal cavity, where the virus was initially delivered. Furthermore, the data support the hypothesis that the dramatic increase in cell abundance in the spleen at day 4 (Figure 1) is most likely the result of very rapid local cell division, rather than the abrupt influx of cells that had multiplied in other locations.

### Memory T cell accumulation is delayed for several days post-infection

**Figure 3:**
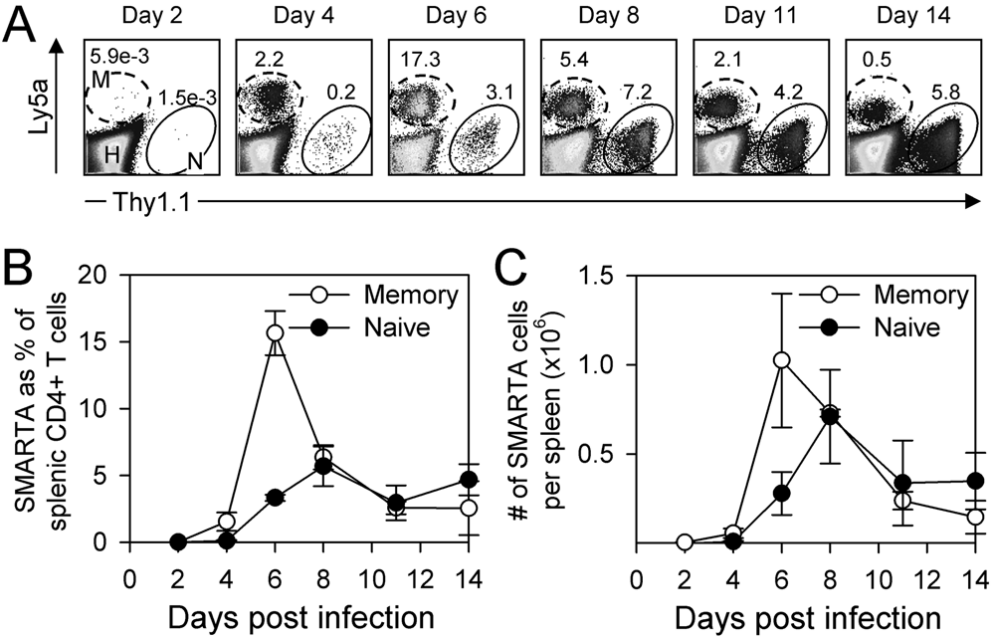
Kinetics of naive and memory CD4^+^ T cells in the same mouse. Wildtype mice containing 1.3×10^3^ naïve SMARTA (Thy1.1) and 1.3×10^3^ memory SMARTA (Ly5a) cells were given LCMV, and the relative abundance of the two SMARTA cell populations was determined by flow cytometry at various times post infection (two mice per time point). A. After gating on CD4^+^ T cells, the host CD4^+^ T cells (H), and the naïve and memory SMARTA cells (N & M respectively) were distinguished by Thy1.1 and Ly5a staining. The numbers indicate the frequencies of naïve and memory SMARTA cells as a percentage of all CD4^+^ T cells. B. The average±SE of the percentage of each population among all CD4^+^ T cells is shown over time. C. The total number of memory or naïve SMARTA CD4^+^ T cells per spleen is shown (average±SE).

Having established the kinetics of naïve T cell division and accumulation, we next evaluated these issues for memory T cells. Memory T cells protect against re-infection better than naïve cells of the same epitope specificity, and several reasons have been advanced to explain this. Memory cells are: (i) are more numerous; (ii) are thought to initiate cell division more quickly; and (iii) express their effector functions more rapidly and in response to lower amount of epitope [3], [10]–[12]. The great majority of comparisons of naïve and memory cells have been carried out in separate mice (naïve & immune mice, respectively). However, such a comparison is complicated by several confounding factors. The abundance of memory cells in immune mice is far greater (at least 1000-fold) than the abundance of the equivalent naïve T cells in naïve mice; consequently, it is not easy to compare the relative changes in cell number between these two populations early after infection. Furthermore, the context within which memory T cell responses are measured (an immune mouse) differs in several ways from that in which primary T cell responses are measured: antigen-presenting cell number and quality will differ (immune mice may contain numerous memory B cells); immune mice may contain preexisting antibody that could facilitate the uptake of viral antigen and lead to quicker processing and presentation of virus-derived peptide by dendritic cells; and the memory cells in immune mice could affect the quantity and distribution of viral antigen. We chose to avoid these confounders, and to directly compare the rates of accumulation of naive and memory T cells in the same mice, by pooling equal numbers of naïve and memory SMARTA CD4^+^ T cells, and transferring them to naïve mice. The mice then were infected with LCMV, and the abundance of naïve and memory cells after infection was followed in the spleen by flow cytometry. The proportions of both T cell populations were similar at day 2 and were near the limits of detection (Figure 3A, representative data from single mice, shown as a percentage of all CD4^+^ T cells). Cumulative proportional data for several mice at each time point are plotted graphically in Figure 3B. T cell accumulation became apparent by day 4 for both naïve and memory populations, but memory cells showed a more dramatic increase at this early time. The increase in the frequency of both populations continued; the memory cell response peaked at day 6 and the naive T cell response peaked at day 8. Memory cell contraction was profound by day 8, whereas the primary effector contraction phase was not yet evident. The same pattern was seen when the absolute numbers of naïve and memory SMARTA CD4^+^ T cells per spleen were evaluated (Figure 3C). Early on, the number of secondary effectors remained relatively unchanged and was similar to the number of primary effector CD4 T cells until day 4. After day 4, the secondary effectors reached a peak that was higher than, and occurred earlier than, that reached by the primary effector response.

### CD4^+^ memory T cells do not initiate division until 3–4 days after infection

**Figure 4:**
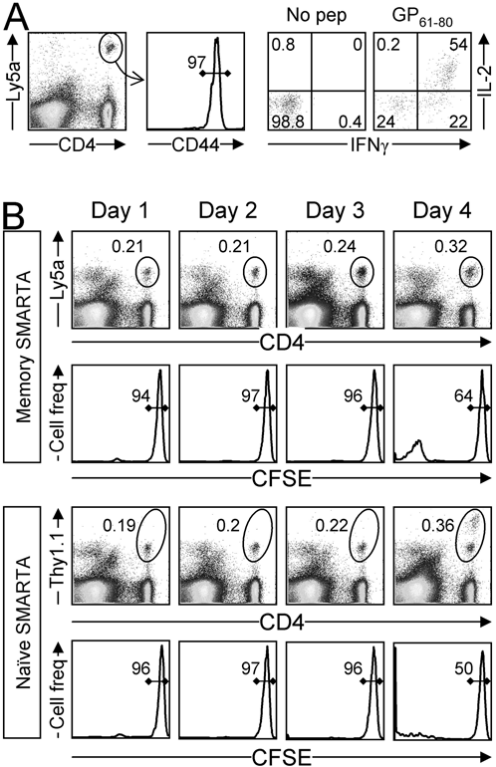
Naive and memory CD4^+^ T cells show near-identical delays in onset of division. Mice containing 2×10^3^ naive SMARTA CD4^+^ T cells (Ly5a) were infected with LCMV and allowed to become immune. A. Six months after infection, memory SMARTA CD4^+^ T cells were isolated from the spleen and analyzed by flow cytometry. The first dot plot identifies the memory SMARTA CD4^+^ T cells (oval). After gating on these cells, the histogram shows their expression of CD44, and the remaining two dot plots evaluate IFNγ and IL-2 production after brief *in vitro* stimulation with GP_61–80_ peptide. B. The memory SMARTA cells (Ly5a) were mixed with naive SMARTA cells (Thy1.1), labeled with CFSE, and then transferred to naive mice. The recipient mice were given approximately 5×10^4^ memory SMARTA CD4 T cells and 5×10^5^ naive SMARTA CD4 T cells. 3 days later, the recipients were infected with LCMV. The dot plots show spleen cells isolated from recipient mice at the indicated times after infection, and the ovals identify the memory SMARTA CD4 T cells (top two rows) and the naive SMARTA CD4 T cells (bottom two rows). The histograms show the CFSE fluorescence of the SMARTA cells, and the numbers in the histograms indicate the percentage of SMARTA CD4^+^ T cells that have not divided. Data are representative of two independent experiments.

The small number of transgenic cells transferred in the preceding experiment prevented our analyzing the very early (day 1) antiviral responses of naïve and memory cells. To more precisely compare the time of onset of cell division in naïve and memory T cells, a larger number (see Materials) of memory SMARTA cells and naïve SMARTA cells were mixed, labeled with CFSE and given to mice; after several days, the recipient mice were given LCMV. Before pooling the cells, we considered it important to demonstrate the authenticity and homogeneity of the memory SMARTA CD4^+^ T cells. To this end, aliquots of the memory cells were evaluated for their *in vivo* responsiveness to peptide antigen, and for the expression of memory markers (Figure 4A); the cells were CD44^hi^, and the majority produced both IL-2 and IFNγ in response to stimulation with cognate peptide (GP_61–80_). After pooling these cells with naïve SMARTA cells, CFSE labeling, inoculation into recipient mice, and infection, the abundances of naïve and memory cells in the same mice were measured daily by flow cytometry (Figure 4B). In this experiment, naïve and memory SMARTA cells began cell division after day 3 and began to accumulate at day 4. These data imply that, when naive and memory T cells are exposed to the same environment, they both show an approximate 3-day delay after infection, before they initiate cell division.

### Onset of division of naïve and memory CD4+ T cells is delayed and synchronous in several lymphoid and non-lymphoid tissues

**Figure 5:**
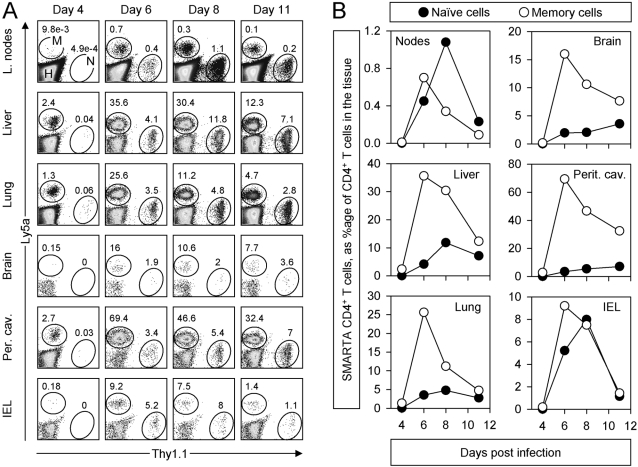
Delayed accumulation of naive and memory CD4^+^ T cells occurs also in non-lymphoid tissues. Mice containing 1.3×10^4^ naïve (Thy1.1) and 1.3×10^4^ memory (Ly5a) SMARTA CD4^+^ T cells were infected with LCMV and, at the indicated times after infection, lymphocytes from several lymphoid and non-lymphoid tissues were isolated and analyzed by flow cytometry. A. Dot plots show gated CD4^+^ T cells isolated from the tissues. The ovals identify the SMARTA cells (N, M  =  naïve & memory respectively), and the numbers indicate their percentage among all CD4 T cells (H  =  host CD4^+^ T cells). B. For each tissue, naïve and memory SMARTA CD4^+^ T cells are shown as percentages of all CD4^+^ T cells (two mice per time point). Note that both naïve and memory cells become prominent after day 4; however, the memory cells dominate the response in the non-lymphoid tissues.

The trafficking pattern of memory T cells differs from that of naive T cells; both CD8 and CD4 memory T cells more readily percolate through non-lymphoid tissues, whereas naive T cells are more restricted to the lymphoid organs [5], [13]–[16]. Therefore, the abundance of naive and memory T cells was followed in lymphoid and non-lymphoid sites at early times after LCMV infection (Figure 5). Equal numbers of naive and memory T cells were pooled, and a low number of pooled cells was administered to naïve mice. The number of cells in recipient mice more closely resembled the endogenous number of naive T cell precursors; however, using this initial low frequency of cells makes it difficult to identify the cells by flow cytometry during the first few days. Therefore, analyses were done starting at day 4 after infection, which is when the upsurge in the number of cells in the spleen begins. At day 4, a few memory SMARTA CD4^+^ T cells could be detected in the lymph node, liver, lung, peritoneal cavity, and IEL, and they were somewhat more abundant than naive T cells (Figure 5A). These observations are consistent with other reports that have shown extensive memory cell or secondary effector cell movement through non-lymphoid tissues. Primary T cells were found in some non-lymphoid sites (liver, lung, peritoneal cavity), but not in others (brain and IEL), probably due to the very low number of naive precursors that were initially given and the limited expansion of naïve cells at this early time point (see Figure 1–[Fig ppat-1000041-g002] Figure 3). However, by day 6, both primary and secondary effector T cell populations showed dramatic increases in number in all of the locations analyzed. The secondary effector response peaked at day 6, and declined in frequency by day 8, whereas the primary effector cells peaked at this time in most locations, except in the brain and peritoneal cavity, where there appeared to be more cells at day 11. When shown graphically as the average percentage of SMARTA CD4^+^ T cells among all infiltrating/resident CD4 T cells, the memory SMARTA CD4^+^ T cell population (open circles, Figure 5B) showed a dramatic increase after day 4 and peaked at day 6; the naive CD4 T cells (closed circles) began to accumulate at the same time, but did so more slowly, and peaked later and at a lower percentage in most sites. It is noteworthy that even in the peritoneal cavity, where the virus was originally delivered – and where, one would expect, viral antigen would be expressed early and in quantity – the pattern of T cell accumulation resembled that seen in the spleen in terms of initial kinetics and the dominance of the memory cells, which argues that the spleen is a good “window” to view the entire immune response to LCMV. Taken together, these data indicate that the primary and secondary T cell responses to LCMV are organism-wide rather than localized, as seen during other infections [15],[17]. Furthermore, the time it takes to initiate cell division in the secondary and primary populations is similar in all organs analyzed, which suggests that, as for primary effector cells (Figure 2) the sudden accumulation of secondary effector cells in the spleen represents abrupt organism-wide cell division rather than selective recruitment to the spleen of recently-divided cells.

### Delayed memory cell division cannot be attributed to the absence of stimulatory antigen in vivo

**Figure 6:**
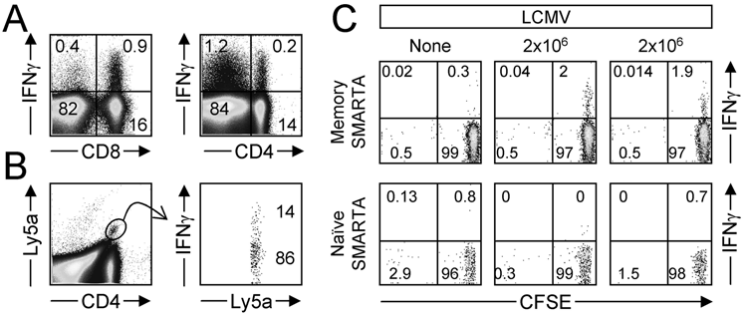
Viral epitopes are presented within hours of infection, and stimulate memory T cell effector functions. Mice that contained approximately 3×10^3^ SMARTA/Ly5a CD4^+^ T cells were infected with LCMV and, 354 days later, were re-challenged intraperitoneally with 2×10^6^ PFU LCMV-Armstong. Six hours post-infection, the mice were given 0.25 mg Brefeldin A i.v., and 6 hours later the spleens were harvested and immediately surface stained for CD4, Ly5a, or CD8, then permeabilized and stained for intracellular IFNγ. The cells were not re-stimulated *ex vivo* with peptide antigen. A. ∼5% of all CD8^+^ T cells, and ∼1% of all CD4^+^ T cells, are actively producing IFNγ in response to infection. B. Using the SMARTA cells transferred ∼1 year previously as an indicator of the responsiveness of virus-specific CD4^+^ memory T cells, ∼14% of LCMV-specific CD4^+^ memory T cells actively produce IFNγ within 12 hours of virus infection. Data shown are from an individual mouse, and are representative of independent datasets. C. A separate set of naive mice were given CFSE-labeled pooled SMARTA cells (4×10^5^ naive SMARTA/Thy1.1 cells and 2×10^4^ memory SMARTA/Ly5a T cells). 4 days later, some of the recipient mice were given LCMV. Six hours later, BFA was administered to all mice, and after a further 6 hours splenocytes were harvested. The cells were immediately stained (without peptide re-stimulation) for CD4, Thy1.1, Ly5a and IFNγ, and were analyzed by flow cytometry. Approximately 2% memory SMARTA cells had begun to synthesize IFNγ in response to LCMV infection (top row) but none of those responding memory cells showed any dilution of CFSE signal. The naïve SMARTA cells (bottom row) failed to produce IFNγ at this early time point post-infection, and no sign of cell division was seen. Data are from one of two independent experiments.

Conceptually, the delay in T cell division, shown above for both naïve and memory T cells, could be regulated by exogenous factors, or could be intrinsic to the T cell. For example, the lack of proliferation at 2 days post infection might reflect an insufficiently prepared microenvironment, e.g., low antigen load; perhaps it takes some time for *in vivo* antigen levels to rise sufficiently to trigger T cells. Thus, we evaluated the ability of T cells to respond to *in vivo* contact with authentic viral antigen at very early times (hours) post-infection, using an approach that we have recently developed; the inoculation of brefeldin A (BFA) into virus-infected mice allows responding T cells to be detected by staining directly *ex vivo* (without *ex vivo* stimulation with synthetic peptide) [18],[19]. CD8^+^ memory cells constitute ∼10% of all CD8^+^ T cells in LCMV-immune mice, and we have previously shown that ∼50% of these virus-specific memory CD8^+^ T cells (i.e., ∼5% of all CD8^+^ T cells in an LCMV-immune mouse) produce IFNγ within 6–12 hours of LCMV infection [18]. Here, we extend the analysis to CD4^+^ T cells. Naïve mice that contained ∼3×10^3^ SMARTA CD4^+^ T cells (Ly5a) were infected with LCMV. 354 days later, the mice were re-infected with virus and 6 hours thereafter were injected with BFA. In these immune mice, approximately 5% of all CD8^+^ T cells had made IFNγ within 12 hours of re-infection with LCMV, recapitulating published data from this laboratory [18], and others [20]. In addition, approximately 1% of all splenic CD4^+^ T cells were IFNγ^+^ at 12 hours after infection (Figure 6A). Thus, memory CD4^+^ T cells, like memory CD8^+^ T cells, elaborate IFNγ within hours of secondary viral infection. To quantify the fraction of CD4^+^ memory T cells of known LCMV specificity that makes IFNγ immediately after infection, the Ly5a SMARTA CD4^+^ T cells were gated (Figure 6B, left dotplot) and their production of IFNγ was determined (right dotplot). 14% of the virus-specific CD4^+^ memory T cells had responded within hours of infection. These data indicate that, within a few hours after in vivo infection, sufficient levels of epitope are presented by both MHC class I and MHC class II to stimulate virus-specific memory CD4^+^ and CD8^+^ T cells to produce IFNγ. This approach allows us to identify the T cells that are actively responding to viral antigen, and therefore permits us to determine if such cells may be undergoing a proliferative response. Therefore, to directly examine whether the cells that are actively making IFNγ immediately after challenge might be undergoing cell division, CFSE-labeled memory and naive SMARTA CD4^+^ T cells were pooled and co-transferred into naive mice. Some of these recipients were infected with LCMV and, 6 hours later, all mice were inoculated with BFA. Data for one uninfected mouse, and two infected mice, are shown in Figure 6C. Within 12 hours after infection of these naïve mice, approximately 2% of the memory SMARTA CD4^+^ T cells were actively producing IFNγ, but those responding cells showed no CFSE dilution. The naive SMARTA cells did not produce IFNγ immediately after virus infection, nor did they undergo cell division. These data confirm a functional difference between naive and memory T cells: only memory T cells rapidly make IFNγ within 12 hours of virus infection [18],[21]. Most important for the present study, the data also show that, very soon after infection, virus-derived peptides are presented to T cells at sufficient levels to induce memory cells to produce IFNγ, yet the cells do not initiate division.

### Changing the microenvironment reduces the length of the lag phase

**Figure 7:**
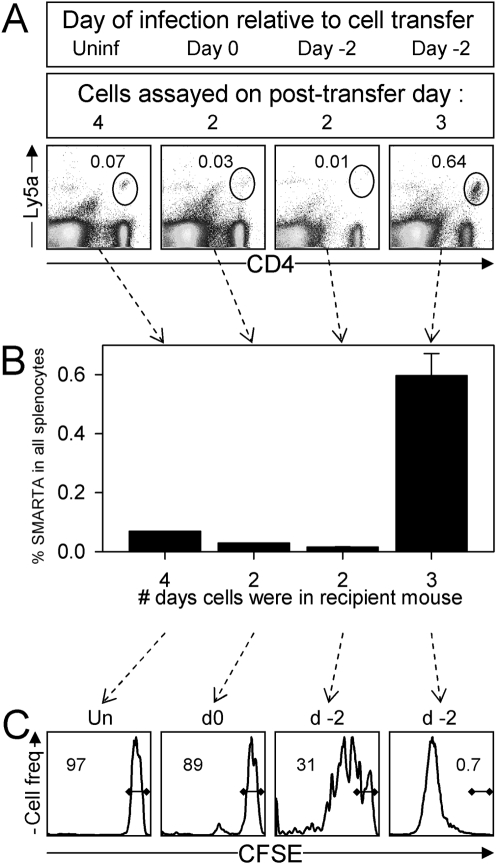
Changing the microenvironment reduces the in vivo delay in T cell division. Naive SMARTA cells were CFSE-labeled and transferred either to mice that had been infected with LCMV two days previously, or to uninfected mice some of which were immediately infected with LCMV. A. 2, 3 or 4 days after cell transfer (as indicated), the spleens of the recipient mice were isolated and the donor SMARTA CD4^+^ T cells were identified by flow cytometry (ovals). Individual mice are shown, and the numbers indicate the proportion of SMARTA cells as a percentage of all spleen cells. Mouse numbers in each of the 4 groups: 1, 1, 3, 3. B. The bar graph shows cumulative data, as percentages of SMARTA CD4^+^ T cells. C. The histograms show the CFSE fluorescence of the indicated SMARTA CD4^+^ T cells. Note that the 3-day delay in proliferation is shortened to 2 days if the mice were pre-infected.

The above data suggest that, within a few hours of virus infection, sufficient antigen is presented by MHC class II to trigger CD4^+^ T cell responses. Thus, we considered the possibility that the lag phase in naïve and memory cell division might result from an intrinsic “brake” that restrains cell proliferation for 2–3 days after antigen contact. *In vitro* analyses argue against this, because T cells cultured with anti-CD3-coated plates or with peptide-loaded DC proliferate by 48 hours [22],[23]. However, *in vitro* analyses are carried out under conditions in which T cells are removed from their normal anatomical and physiological relationships and, for this reason, it is important to evaluate the issue *in vivo*. To do this, CFSE-labeled naïve SMARTA cells were transferred into mice that had been pre-infected with LCMV, and therefore contained a microenvironment that was well-prepared for supporting the initiation of T cell division. From our data in Figure 1, we knew that T cells began to divide around 3 days post-infection, suggesting that at ∼day 2 p.i. the local microenvironment was supportive. Therefore, mice were infected with LCMV, and 2 days later they received naïve CFSE-labeled SMARTA CD4^+^ T cells. Some recipient mice were left uninfected, and others were given virus on the day of cell transfer. The transferred cells were assayed on days 2, 3 or 4 post-transfer (Figure 7). If the naïve SMARTA CD4^+^ T cells were transferred into an uninfected mouse that remained uninfected, cell numbers remained low for at least 4 days (Figure 7A & B, first column) and CFSE remained undiluted (Figure 7C). If the cells were transferred into mice that were concurrently infected (Figure 7, second column), there was neither accumulation nor CFSE dilution at 2 days post transfer, recapitulating the data in Figure 4. In contrast, if the cells were transferred into mice that had been infected two days previously (Figure 7, column 3) then, at the same time point post-transfer (2 days) there was readily-detectable CFSE dilution, indicating that the local environment can exert a substantial effect on the onset of T cell division. This is highlighted by the explosive proliferation that was observed in mice that had been pre-infected, and in which the transferred cells were allowed to incubate for 3 days (Figure 7, column 4); in pre-infected mice, the number of SMARTA cells increased ∼40-fold between day 2 and day 3. Taken together, our data indicate that there is sufficient antigen present within hours of infection to trigger CD4^+^ T cell responses (Figure 6), but that critical changes in the host microenvironment occur around day 2/3 post-infection that allow virus-specific CD4^+^ T cells to initiate their proliferation (Figure 7). These data confirm that the first response of memory T cells, when re-exposed to infection, is to produce IFNγ but not to divide, which is consistent with other reports [1], [21], [24]–[26].

## Discussion

Earlier analyses examining primary and secondary CD8^+^ T cell responses in the same mouse after live microbial infection showed that memory T cells accumulate faster than naïve T cells, but that both populations reached their numerical peak at approximately the same time [27]–[29]. One hypothesis proposed to explain the more rapid increase in CD8^+^ memory T cell numbers was that, after antigen contact, memory T cells initiate cell division more quickly; data supporting this idea has been reported not only for CD8^+^ T cells [1] but also for CD4^+^ T cells [2]. However, other *in vitro* investigations have indicated that naïve and *in vivo*-primed memory T cells initiate proliferation at a similar time point after antigen exposure [26],[29]. Additional analyses of *in vivo* CD8^+^ T cell responses to live microbial infection have reported differences in abundance between primary and secondary (memory) T cells [27],[28] but these studies examined later time points after infection, and thus could not distinguish between, for example, different times of onset of cell division and different trafficking patterns, which are known to differ between memory and naïve T cells [5],[13]. Indeed, the difference in anatomical distribution of naïve and memory cells could be relevant to the time of onset of cell division because one population (presumably, the memory population) might encounter antigen sooner after infection, as has been proposed for some respiratory tract infections [15], [30]–[34]. Therefore, although it is clear that the acquisition of memory T cells is beneficial to the host, the underlying reason(s) for the “superiority” of memory cells, compared to naïve cells, remains obscure. In this study, we asked: how do naive and memory T cells in lymphoid and non-lymphoid tissues respond in the days immediately following a live, systemic, viral infection?

The principal conclusions from our study are that: (i) in a virus-infected animal, both naïve and memory CD4^+^ T cells show a similar and extended delay of ∼72 hours before they begin to divide; (ii) this is true in both lymphoid and non-lymphoid tissues; and (iii) this *in vivo* delay occurs despite viral antigen reaching T cell-stimulatory levels within 6–12 hours of infection. A lag phase prior to the onset of CD8^+^ T cell proliferation has been previously reported in a non-infectious model system, in which cells were transferred into immunodeficient mice; proliferation of HY-specific CD8^+^ T cells was not immediate, and memory cells showed a shorter delay (∼8 hours) compared to naive T cells (∼24–48 hours) [1]. Our study differs in several ways. First, we use an infectious model, and immunocompetent mice. Second, for both CD4^+^ and CD8^+^ T cells, we observe that the delay in proliferation is 48–72 hours and, third, the delay is the same for both memory and naïve cells. The observation that naive and memory T cells initiate cell division concurrently is consistent with the data reported in other models [25],[26],[29],[35]. Cell division is, of course, a complex and lengthy process, and our experimental approach using CFSE measures the final phase: physical separation of the cell membranes and consequent dilution of the dye. We cannot conclude, from our data, that the molecular events that precede cell division (e.g., DNA replication) are initiated concurrently in naïve and memory CD4^+^ T cells. Our *in vivo* data support recent *in vitro* findings, which showed that naive and memory transgenic CD8^+^ T cells initiate cell division at the same time [29]. So, while other investigators have shown a lag in antigen-driven and antigen-independent T cell proliferation [22], we show that this lag occurs *in vivo* and in the context of live systemic virus infection. We also extend this to memory T cells. Consistent with some studies [27]–[29], we see a more robust increase in the number of memory T cells, and we extend this finding by showing that this is not attributable to earlier onset of cell division; both naïve and memory T cells initiate division concurrently. Moreover, LCMV induces a systemic response where T cell responses occur simultaneously (Figure 2); hence, the lag is not related to the movement of cells or their initial presence at sites of infection. Our data confirm and extend some earlier reports and suggest that the “faster” memory T cell response reported in many models is due neither to their more rapid initiation of proliferation, nor to their more rapid division rate [1],[2]; instead, the more robust accumulation of memory cells, observed in both lymphoid and non-lymphoid organs [5],[13], more likely results from their higher precursor frequency at the time of infection, perhaps combined with enhanced survival during the early proliferative response.

What factors might regulate the delay in, and the ultimate onset of, T cell division? It is particularly striking that, in a virus-infected host, CD4^+^ memory T cells express their effector functions within hours of infection (Figure 6), but fail to divide for several days (Figures 3, 4, 5). One explanation for this phenomenon is that more antigen is required to trigger cell division than is needed to drive cytokine synthesis, and that this higher threshold is reached only at 2–3 days post infection. However, memory T cells are more sensitive than naive T cells to antigen and thus, if this argument were valid, memory cells should initiate proliferation sooner; yet they do not. Other analyses indicate that antigen dose affects the number of cells that are recruited into the proliferative response, but not the time when proliferation begins [22],[36]. As an alternative to antigen levels, one can imagine that the acquisition of key costimulatory molecules by dendritic cells might govern the onset of naive and memory T cell division. There is evidence that some costimulatory molecules are expressed in a particular order, which could orchestrate this early T cell stage [37],[38], and recent data suggest that B7/CD28 signaling thresholds are instrumental in regulating cell cycle progression in T cells [36].

The above explanations for the T cell lag phase invoke the early absence of positive factors – for example, insufficient antigen or co-stimulatory molecules. However, it is equally possible that the delay may reflect active negative regulation of T cells by host factors; releasing this brake allows division to begin. Under this scenario, the demonstration of immediate T cell proliferation using *in vitro* studies can be criticized because the use of disrupted tissues might abrogate such negative regulatory interactions, particularly if they require spatial organization. For example, the delay in LCMV-specific T cell proliferation reported herein coincides with peak NK cell activities in this model [39]; one can speculate that, in intact tissues, there may be cellular restraints such as the local consumption of key growth factors, or the local expression of inhibitory cytokines, by NK cells [40]. Furthermore, regulatory T cells, which are present throughout the body, also may constrain T cell proliferation through the production of cytokines that impede T cell responses, such as IL-10 and TGFβ. Changes in the local microenvironment could occur in response to inflammation and perhaps lead to particular metalloprotease activity, and relieve T cells of LAG-3-mediated suppression of proliferation [41]. It has been proposed that early negative regulation may be imposed by early inflammation, in particular by the interferons: we consider this unlikely, because direct IFNγ signals enhance the expansion of CD8^+^ and CD4^+^ T cells [42],[43], and a similar effect has been described for direct IFNαβ signals [44],[45]. If such negative regulatory mechanisms are involved, then it is interesting to speculate that there may be microbes that can engage the brakes in the immune response, thus leading to a delayed immune response, which would enable the pathogen to complete its replication cycle or to spread to a new host. It is noteworthy that some infectious agents induce T cell responses that are much delayed in comparison to that mounted against LCMV; for example, the peak responses against some gamma herpesviruses [46], Histoplasma [47], and mycobacteria [48],[49] occur two weeks or more after infection.

Naïve and memory cells are equivalent in their lag phase but, once proliferation begins, memory cells rapidly outstrip their naïve counterparts (Figure 3) in most anatomical sites (Figure 5). Memory cells outperform naïve cells in several ways, and it is possible that, in the mice containing both memory and naive SMARTA CD4^+^ T cells, the memory cells out-compete the naive T cells for limiting amounts of cytokine, thereby slowing the expansion of the primary T cell response [1]. The memory T cells might occlude naive T cell responses, possibly by associating very closely with APCs and impeding naïve cell access to these cells [50],[51]. However, other investigators have shown that even during an ongoing recall response, naive T cells can be recruited, suggesting that competition by memory cells, if present, must be incomplete [28],[52],[53]. It is tempting to conclude that the more rapid increase in memory cell numbers must result from these cells' having a shorter division time, but recent analyses have shown that naive and memory T cells divide at the same rate [29]. Thus it is possible that the numerical difference between memory and naïve cells can, at least in part, be attributed to better survival of daughter memory cells. Other investigators have shown that T cell apoptosis occurs throughout the expansion phase, and that much of this is due to caspase activity [54]. Memory T cells express greater amounts of bcl2 and are protected from apoptosis, and secondary effector cells show a protracted contraction phase and less overall cell loss [1], [27], [28], [55]–[57]. Hence, the more robust early accumulation of memory T cells may be due to their improved survival, but not to faster cell division.

What are the evolutionary benefits of a delay in antiviral T cell division, given that CD8^+^ and CD4^+^ T cells are essential for eliminating most virus infections and for driving other immune responses? Perhaps the expression of effector functions and cell division are mutually exclusive: the immediate onset of cell division might preclude the memory cells' expressing their cytokines, thereby preventing optimal early control of the infection. Alternatively, if memory cell numbers rose precipitously immediately following infection, they might suppress the recruitment of naïve T cells of different epitope specificity; thus, by delaying the expansion of memory cells, the host may ensure the diversification of the microbe-specific T cell response. Such diversification would, presumably, be beneficial for combating the microbial variants that inevitably emerge. It also is possible that the lag phase represents a period of time during which the innate response, and the effector functions of the memory T cell response, are given an opportunity to quickly control the infection. If this is successful, then the onset of memory T cell division will take place in a relatively non-inflammatory microenvironment, and thereafter will proceed only to a limited extent. Conversely, if the immune system's early attempt to control the infection fails, then T cell division will begin in a more pro-inflammatory microenvironment – which will include abundant type I and type II interferons – and so the T cell response will be driven to a higher peak. In this way, the T cell response escalates most when the infection cannot be resolved within the first few days. Given that T cells are capable of such explosive proliferation, this mechanism may reduce the risk of unwanted immunopathology, including autoreactive T cell responses.

## Materials and Methods

### Mice and virus

C57BL/6 mice were purchased from The Scripps Research Institute (TSRI) breeding facility. C57BL/6 mice congenic for Thy1.1 (B6.PL-Thy1^a^/CyJ) were purchased from The Jackson Laboratory. SMARTA TCR-transgenic mice specific for the I-A^b^ LCMV epitope GP_61–80_ [58] were crossed to C57BL/6.Ly5a mice (B6.SJL-Ptprc^a^Pep3^b^/BoyJ) to generate SMARTA.Ly5a mice or to B6.PL-Thy1^a^/CyJ mice to generate SMARTA/Thy1.1 mice [6],[43]. P14 TCR-transgenic mice specific for the LCMV epitope GP_33–41_ [59] on the H-2^b^ background were crossed to B6.PL mice to generate the P14/Thy1.1 strain. Mice were infected by i.p. administration of 2×10^5^ plaque forming units of LCMV (Armstrong strain). Quantitation of virus in the tissues was done by plaque assay on Vero cell monolayers. All experiments were approved by the TSRI Animal Care and Use Committee.

### Isolation of lymphocytes

Spleen cells and lymph node cells (mix of inguinal, brachial, and axillary nodes) were prepared using standard protocols, with red blood cell lysis. Lymphocyte isolation from other tissues was done as previously described [14]. Mice were first perfused with PBS through the heart. The liver was additionally perfused directly by injecting PBS through the hepatic artery. The lungs and small intestine (with the Peyer's patches removed) were minced and digested with collagenase. The liver and brain were triturated in a Dounce homogenizer to make a cell suspension. Lymphocytes were separated from the rest of the tissue cells by resuspending them in 44% Percoll and floating them onto a 56% Percoll cushion, followed by centrifugation. Lymphocytes were isolated at the interface of the two layers.

### Flow cytometry

Spleen cells were stained directly ex vivo with fluorochrome-conjugated anti-CD4 (clone RM4-5), anti-CD8 (clone 53-6.7), anti-Thy1.2 (CD90.2, clone 53-2.1), anti-Thy1.1 (CD90.1, clone HIS51), anti-CD44 (clone IM7), anti-Ly5a (Ly5.1, clone A20) all purchased from eBioscience.com. The staining reaction was done in the presence of unlabeled antibodies against Fc-receptors to block fluorochrome-conjugated antibodies from binding to FcR+ cells; “FcBlock” was purchased from BD-Pharmingen, La Jolla, CA. The intracellular staining assay was performed as described previously [60] using anti-IFNγ (clone XMG1.2), anti-TNF (clone MP6-XT22), and anti-IL-2 (clone JES6-5H4) from eBioscience. Cell staining was analyzed by 4-color flow cytometry using a BD Biosciences FACSCALIBUR and FloJo software (Tree Star, Ashland OR).

### Adoptive transfers

Flow cytometry was used to determine the frequency of transgenic CD4^+^ T cells (Vα2^+^Vβ8.3^+^) among all spleen cells in SMARTA mice or the frequency of transgenic CD8^+^ T cells (Vα2^+^Vβ8.1/2^+^) among all spleen cells in P14 mice. For the majority of experiments, a small number (1–3×10^4^) of transgenic T cells were injected intravenously into recipient mice, and the mice were infected 4–7 days after cell transfer (at which time, given ∼10% “take”, the mice will contain only ∼10^3^ transgenic cells). In the experiments designed to evaluate the very early onset of T cell division, a larger number of transgenic cells (1–10×10^5^) was labeled with 5mM CFSE before transfer into recipient mice. This larger number of transgenic T cells was necessary to allow the cells to be monitored as early as 1 day post infection.

### Using brefeldin A injection to identify T cells that have responded to in vivo antigen contact

As described [18],[19], 250 µg of brefeldin A (Sigma, St. Louis, MO) was injected i.v. into mice, to block the *in vivo* secretion of cytokines. Six hours later, the mice were sacrificed and splenocytes were harvested and immediately surface stained to identify T cells, then permeabilized and stained for intracellular IFNγ . In this assay, the T cells are not exposed to synthetic peptides *ex vivo*.

## References

[1] Veiga-Fernandes H, Walter U, Bourgeois C, McLean A, Rocha B (2000). Response of naive and memory CD8+ T cells to antigen stimulation in vivo.. Nat Immunol.

[2] Rogers PR, Dubey C, Swain SL (2000). Qualitative changes accompany memory T cell generation: faster, more effective responses at lower doses of antigen.. J Immunol.

[3] Slifka MK, Whitton JL (2001). Functional avidity maturation of CD8^+^ T cells without selection of higher affinity TCR.. Nat Immunol.

[4] Lefrancois L (2006). Development, trafficking, and function of memory T-cell subsets.. Immunol Rev.

[5] Weninger W, Crowley MA, Manjunath N, von Andrian UH (2001). Migratory properties of naive, effector, and memory CD8^+^ T cells.. J Exp Med.

[6] Whitmire JK, Benning N, Whitton JL (2006). Precursor frequency, nonlinear proliferation, and functional maturation of virus-specific CD4^+^ T cells.. J Immunol.

[7] Jiang J, Zenewicz LA, San Mateo LR, Lau LL, Shen H (2003). Activation of antigen-specific CD8 T cells results in minimal killing of bystander bacteria.. J Immunol.

[8] Maxwell JR, Rossi RJ, McSorley SJ, Vella AT (2004). T cell clonal conditioning: a phase occurring early after antigen presentation but before clonal expansion is impacted by Toll-like receptor stimulation.. J Immunol.

[9] Carrero JA, Calderon B, Unanue ER (2004). Type I interferon sensitizes lymphocytes to apoptosis and reduces resistance to Listeria infection.. J Exp Med.

[10] Seder RA, Ahmed R (2003). Similarities and differences in CD4+ and CD8+ effector and memory T cell generation.. Nat Immunol.

[11] Kersh EN, Fitzpatrick DR, Murali-Krishna K, Shires J, Speck SH (2006). Rapid demethylation of the IFN-gamma gene occurs in memory but not naive CD8 T cells.. J Immunol.

[12] Slifka MK, Whitton JL (2000). Activated and memory CD8^+^ T cells can be distinguished by their cytokine profiles and phenotypic markers.. J Immunol.

[13] Masopust D, Vezys V, Usherwood EJ, Cauley LS, Olson S (2004). Activated primary and memory CD8 T cells migrate to nonlymphoid tissues regardless of site of activation or tissue of origin.. J Immunol.

[14] Masopust D, Vezys V, Marzo AL, Lefrancois L (2001). Preferential localization of effector memory cells in nonlymphoid tissue.. Science.

[15] Roman E, Miller E, Harmsen A, Wiley J, von Andrian UH (2002). CD4 effector T cell subsets in the response to influenza: heterogeneity, migration, and function.. J Exp Med.

[16] Wherry EJ, Teichgraber V, Becker TC, Masopust D, Kaech SM (2003). Lineage relationship and protective immunity of memory CD8 T cell subsets.. Nat Immunol.

[17] Mayer KD, Mohrs K, Crowe SR, Johnson LL, Rhyne P (2005). The functional heterogeneity of type 1 effector T cells in response to infection is related to the potential for IFN-gamma production.. J Immunol.

[18] Liu F, Whitton JL (2005). Cutting Edge: Re-evaluating the *in vivo* cytokine responses of CD8^+^ T cells during primary and secondary viral infections.. J Immunol.

[19] Foster B, Prussin C, Liu F, Whitmire JK, Whitton JL, Coligan JE, Kruisbeek AM, Marguiles DH, Shevach EM, Strober W (2007). Detection of Intracellular Cytokines by Flow Cytometry.. Current Protocols in Immunology.

[20] Mempel TR, Henrickson SE, von Andrian UH (2004). T-cell priming by dendritic cells in lymph nodes occurs in three distinct phases.. Nature.

[21] Tebo AE, Fuller MJ, Gaddis DE, Kojima K, Rehani K (2005). Rapid recruitment of virus-specific CD8 T cells restructures immunodominance during protective secondary responses.. J Virol.

[22] Lee WT, Pasos G, Cecchini L, Mittler JN (2002). Continued antigen stimulation is not required during CD4^+^ T cell clonal expansion.. J Immunol.

[23] Wells AD, Gudmundsdottir H, Turka LA (1997). Following the fate of individual T cells throughout activation and clonal expansion. Signals from T cell receptor and CD28 differentially regulate the induction and duration of a proliferative response.. J Clin Invest.

[24] Lalvani A, Brookes R, Hambleton S, Britton WJ, Hill AV (1997). Rapid effector function in CD8^+^ memory T cells.. J Exp Med.

[25] Cho BK, Wang C, Sugawa S, Eisen HN, Chen J (1999). Functional differences between memory and naïve CD8 T cells.. Proc Natl Acad Sci U S A.

[26] Zimmermann C, Prevost-Blondel A, Blaser C, Pircher H (1999). Kinetics of the response of naive and memory CD8 T cells to antigen: similarities and differences.. Eur J Immunol.

[27] Grayson JM, Harrington LE, Lanier JG, Wherry EJ, Ahmed R (2002). Differential sensitivity of naive and memory CD8+ T cells to apoptosis in vivo.. J Immunol.

[28] Badovinac VP, Messingham KA, Hamilton SE, Harty JT (2003). Regulation of CD8^+^ T cells undergoing primary and secondary responses to infection in the same host.. J Immunol.

[29] Stock AT, Jones CM, Heath WR, Carbone FR (2006). Cutting edge: central memory T cells do not show accelerated proliferation or tissue infiltration in response to localized herpes simplex virus-1 infection.. J Immunol.

[30] Hogan RJ, Usherwood EJ, Zhong W, Roberts AA, Dutton RW (2001). Activated antigen-specific CD8^+^ T cells persist in the lungs following recovery from respiratory virus infections.. J Immunol.

[31] Jelley-Gibbs DM, Brown DM, Dibble JP, Haynes L, Eaton SM (2005). Unexpected prolonged presentation of influenza antigens promotes CD4 T cell memory generation.. J Exp Med.

[32] Roberts AD, Ely KH, Woodland DL (2005). Differential contributions of central and effector memory T cells to recall responses.. The Journal of Experimental Medicine.

[33] Zammit DJ, Turner DL, Klonowski KD, Lefrancois L, Cauley LS (2006). Residual antigen presentation after influenza virus infection affects CD8 T cell activation and migration.. Immunity.

[34] Ely KH, Cookenham T, Roberts AD, Woodland DL (2006). Memory T cell populations in the lung airways are maintained by continual recruitment.. J Immunol.

[35] Garcia S, DiSanto J, Stockinger B (1999). Following the development of a CD4 T cell response in vivo: from activation to memory formation.. Immunity.

[36] Bonnevier JL, Mueller DL (2002). Cutting edge: B7/CD28 interactions regulate cell cycle progression independent of the strength of TCR signaling.. J Immunol.

[37] Bertram EM, Lau P, Watts TH (2002). Temporal segregation of 4-1BB versus CD28-mediated costimulation: 4-1BB ligand influences T cell numbers late in the primary response and regulates the size of the T cell memory response following influenza infection.. J Immunol.

[38] Rogers PR, Song J, Gramaglia I, Killeen N, Croft M (2001). OX40 promotes Bcl-xL and Bcl-2 expression and is essential for long-term survival of CD4 T cells.. Immunity.

[39] Orange JS, Biron CA (1996). Characterization of early IL-12, IFN-α/β, and TNF effects on antiviral state and NK cell responses during murine cytomegalovirus infection.. J Immunol.

[40] Su HC, Nguyen KB, Salazar-Mather TP, Ruzek MC, Dalod MY (2001). NK cell functions restrain T cell responses during viral infections.. Eur J Immunol.

[41] Li N, Wang Y, Forbes K, Vignali KM, Heale BS (2007). Metalloproteases regulate T-cell proliferation and effector function via LAG-3.. EMBO J.

[42] Whitmire JK, Tan JT, Whitton JL (2005). Interferon-γ acts directly on CD8^+^ T cells to increase their abundance during virus infection.. J Exp Med.

[43] Whitmire JK, Benning N, Whitton JL (2005). Cutting Edge: Early IFN-γ signaling directly enhances primary antiviral CD4^+^ T cell responses.. J Immunol.

[44] Havenar-Daughton C, Kolumam GA, Murali-Krishna K (2006). Cutting Edge: The direct action of type I IFN on CD4 T cells is critical for sustaining clonal expansion in response to a viral but not a bacterial infection.. J Immunol.

[45] Kolumam GA, Thomas S, Thompson LJ, Sprent J, Murali-Krishna K (2005). Type I interferons act directly on CD8 T cells to allow clonal expansion and memory formation in response to viral infection.. J Exp Med.

[46] Topham DJ, Cardin RC, Christensen JP, Brooks JW, Belz GT (2001). Perforin and Fas in murine gammaherpesvirus-specific CD8(+) T cell control and morbidity.. J Gen Virol.

[47] Lin JS, Wu-Hsieh BA (2004). Functional T cells in primary immune response to histoplasmosis.. Int Immunol.

[48] van Faassen H, Dudani R, Krishnan L, Sad S (2004). Prolonged antigen presentation, APC-, and CD8+ T cell turnover during mycobacterial infection: comparison with Listeria monocytogenes.. J Immunol.

[49] Dalton DK, Haynes L, Chu CQ, Swain SL, Wittmer S (2000). Interferon gamma eliminates responding CD4 T cells during mycobacterial infection by inducing apoptosis of activated CD4 T cells.. J Exp Med.

[50] Gray PM, Reiner SL, Smith DF, Kaye PM, Scott P (2006). Antigen-experienced T cells limit the priming of naive T cells during infection with Leishmania major.. J Immunol.

[51] Ramakrishna C, Stohlman SA, Atkinson RA, Hinton DR, Bergmann CC (2004). Differential regulation of primary and secondary CD8+ T cells in the central nervous system.. J Immunol.

[52] Turner SJ, Cross R, Xie W, Doherty PC (2001). Concurrent naive and memory CD8(+) T cell responses to an influenza A virus.. J Immunol.

[53] Vezys V, Masopust D, Kemball CC, Barber DL, O'Mara LA (2006). Continuous recruitment of naive T cells contributes to heterogeneity of antiviral CD8 T cells during persistent infection.. J Exp Med.

[54] Grayson JM, Laniewski NG, Lanier JG, Ahmed R (2003). Mitochondrial potential and reactive oxygen intermediates in antigen-specific CD8^+^ T cells during viral infection.. J Immunol.

[55] Badovinac VP, Porter BB, Harty JT (2002). Programmed contraction of CD8^+^ T cells after infection.. Nat Immunol.

[56] Corbin GA, Harty JT (2004). Duration of infection and antigen display have minimal influence on the kinetics of the CD4^+^ T cell response to Listeria monocytogenes infection.. J Immunol.

[57] Grayson JM, Zajac AJ, Altman JD, Ahmed R (2000). Cutting edge: increased expression of bcl-2 in antigen-specific memory CD8^+^ T cells.. J Immunol.

[58] Oxenius A, Bachmann MF, Zinkernagel RM, Hengartner H (1998). Virus-specific MHC-class II-restricted TCR-transgenic mice: effects on humoral and cellular immune responses after viral infection.. Eur J Immunol.

[59] Pircher H, Burki K, Lang R, Hengartner H, Zinkernagel RM (1989). Tolerance induction in double specific T-cell receptor transgenic mice varies with antigen.. Nature.

[60] Whitmire JK, Asano MS, Murali-Krishna K, Suresh M, Ahmed R (1998). Long-term CD4 Th1 and Th2 memory following acute lymphocytic choriomeningitis virus infection.. J Virol.

